# Graphene-clad microfibre saturable absorber for ultrafast fibre lasers

**DOI:** 10.1038/srep26024

**Published:** 2016-05-16

**Authors:** X. M. Liu, H. R. Yang, Y. D. Cui, G. W. Chen, Y. Yang, X. Q. Wu, X. K. Yao, D. D. Han, X. X. Han, C. Zeng, J. Guo, W. L. Li, G. Cheng, L. M. Tong

**Affiliations:** 1 State Key Laboratory of Transient Optics and Photonics, Xi’an Institute of Optics and Precision Mechanics, Chinese Academy of Sciences, Xi’an 710119, China; 2 State Key Laboratory of Modern Optical Instrumentation, Department of Optical Engineering, Zhejiang University, Hangzhou 310027, China

## Abstract

Graphene, whose absorbance is approximately independent of wavelength, allows broadband light–matter interactions with ultrafast responses. The interband optical absorption of graphene can be saturated readily under strong excitation, thereby enabling scientists to exploit the photonic properties of graphene to realize ultrafast lasers. The evanescent field interaction scheme of the propagating light with graphene covered on a D-shaped fibre or microfibre has been employed extensively because of the nonblocking configuration. Obviously, most of the fibre surface is unused in these techniques. Here, we exploit a graphene-clad microfibre (GCM) saturable absorber in a mode-locked fibre laser for the generation of ultrafast pulses. The proposed all-surface technique can guarantee a higher efficiency of light–graphene interactions than the aforementioned techniques. Our GCM-based saturable absorber can generate ultrafast optical pulses within 1.5 μm. This saturable absorber is compatible with current fibre lasers and has many merits such as low saturation intensities, ultrafast recovery times, and wide wavelength ranges. The proposed saturable absorber will pave the way for graphene-based wideband photonics.

Ultrafast lasers that produce pico- to femto-second optical pulses have many potential applications, such as in basic scientific research, materials processing, electronic components, metrology, telecommunication, and medicine^1^,^2^,^3^,^4^,^5^. Currently, the majority of ultrafast lasers employ a saturable absorber (SA) to transform the laser continuous wave (CW) into optical pulse trains^6^,^7^,^8^,^9^,^10^,^11^,^12^,^13^,^14^. Key requirements for such SAs are a fast response time, a broad wavelength range, a low cost, and easy integration into an optical system^1^.

Graphene, a single sheet of carbon atoms forming a honeycomb crystal lattice, exhibits a variety of exceptional electronic and photonic properties^15^,^16^,^17^. The gapless linear dispersion of the Dirac electrons in graphene offers the ideal solution for SAs^6^. Bao *et al*. proposed using an ultrathin graphene sheet as an SA in an ultrafast fibre laser^18^, as shown in Fig. 1(a). Sun *et al*. incorporated graphene flakes into polyvinyl alcohol (PVA) and demonstrated the use of a graphene–PVA nanocomposite film as an SA^6^, as shown in Fig. 1(b). These two types of SAs can be integrated between a pair of fibre connector ends easily. Fibre ferrule-type graphene SAs are widely used to realize passively mode-locked lasers because of their excellent fibre compatibility and flexibility^11^,^19^. The proposed schemes illustrated in Fig. 1(a,b) demonstrate that only pinhole area graphene has been used for SAs. However, this physically touching scheme can cause the distortion and/or damage to the graphene. As an improved scheme, an evanescent field interaction scheme of the propagating light with graphene on a D-shaped fibre has been proposed (Fig. 1(c)). This scheme overcomes the optical power-induced thermal damage and guarantees a strong nonlinear effect from graphene because of the long lateral interaction length^20^,^21^,^22^,^23^. An equivalent method is to replace a D-shaped fibre with a microfibre^24^, as shown in Fig. 1(d). The schemes presented in Fig. 1(c,d) illustrate that only a small area of fibre is used for the interaction of the light with graphene. To increase the interaction area of the light with graphene, a fibre taper embedded in a graphene/polymer composite has been proposed^25^,^26^, as illustrated in Fig. 1(e). This technique is based on earlier work (e.g., Khanh’s scheme^27^), in which a carbon nanotube/polymer composite was used.

To enhance the interaction of graphene with the evanescent field of propagating light, it is imperative to explore new operating schemes of graphene-based SAs with a nonblocking configuration. When a layer of graphene is wrapped around a microfibre (Fig. 2), the all-surface technique can guarantee the maximum efficiency of the nonlinear effect of the graphene. Although this technique was proposed by Li *et al*.^28^, it was used to achieve an all-optical modulator rather than an SA. The length of graphene wrapped around the microfibre was approximately 16 μm. To increase the graphene-cladded length, we propose a new technique, in which the length of graphene is greater than 200 μm (Fig. 3). Subsequently, we propose and demonstrate the use of a graphene-clad microfibre (GCM) as an SA in a mode-locked fibre laser for the generation of ultrafast soliton pulses. The improved saturable absorption properties originate from the enhanced light-graphene interaction due to the optical field confined to the wave-guiding microfibre^29^.

## Results

### GCM saturable absorber

**Figure 2:**
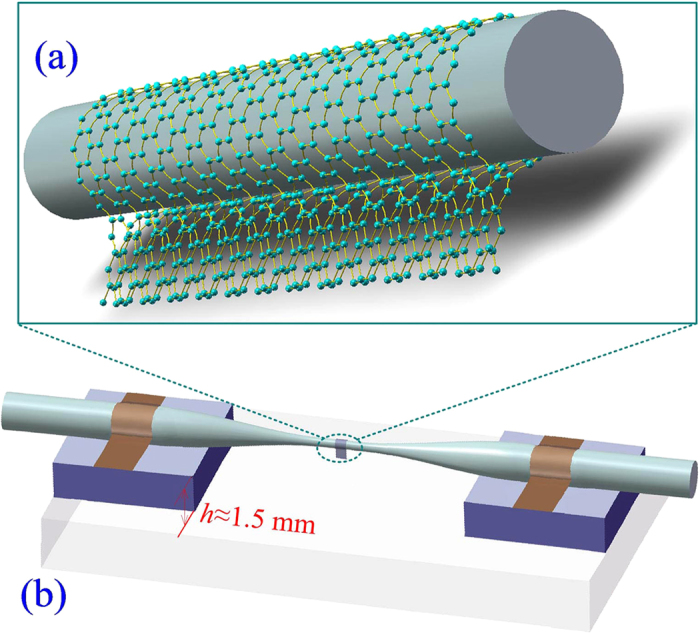
Schematic diagram of (**a**) the GCM SA and (**b**) the hollow substrate together with GCM. A graphene monolayer is wrapped around a microfibre. The GCM SA is fixed onto a hollow substrate with a height of *h* ≈ 1.5 mm.

**Figure 3:**
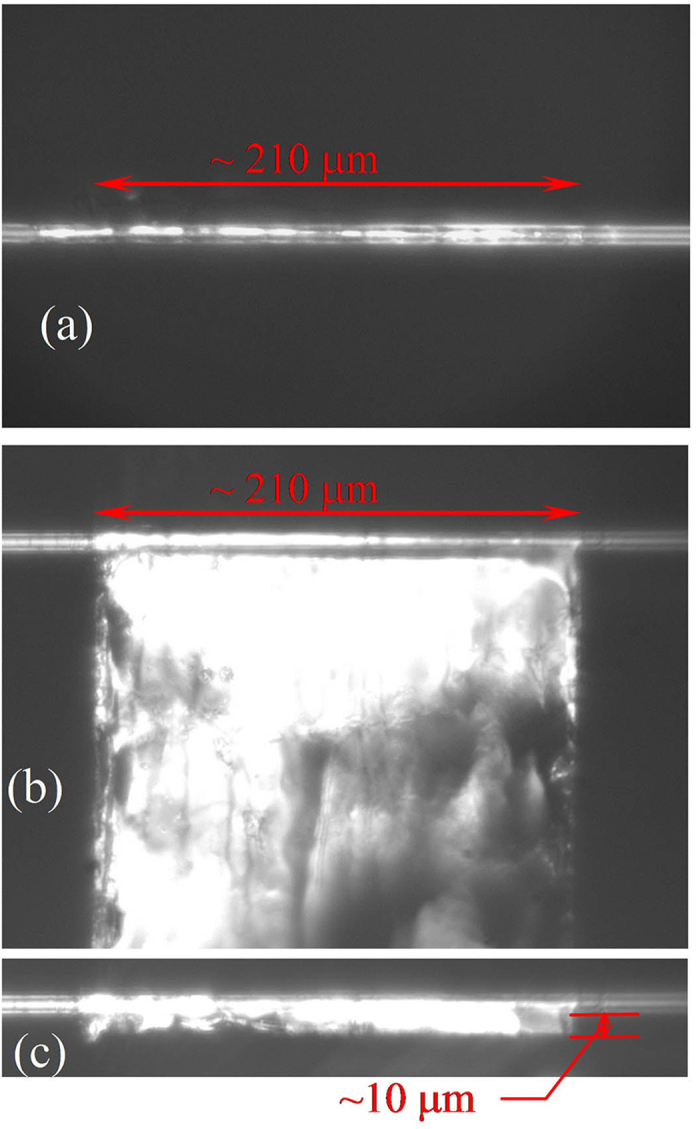
Optical microscope images of a microfibre with the PMMA/graphene. (**a**) Top view before cutting. Lateral view (**b**) before and (**c**) after cutting. The detailed cutting procedure is illustrated in a video (see video: CutGraphene.swf in the online supplementary information).

The microfibre, with a minimum diameter of ~6 μm, is fabricated via a flame-brushing technique and integrated onto a hollow glass substrate, as shown in Fig. 2(b). The monolayer graphene is grown on Cu foil using the chemical vapour deposition (CVD) method^30^. First, the polymethyl methacrylate (PMMA)/graphene sheet is wrapped around the microfibre, as shown in Fig. 3(b). The suspended PMMA/graphene is then cut to a width of ~10 μm alongside the microfibre, as shown in Fig. 3(c). The detailed cutting procedure is illustrated in a video included in the online supplementary information. Finally, the PMMA is removed. As shown in Fig. 2(a), the GCM saturable absorber is realized by wrapping monolayer graphene around a microfibre. The detailed fabrication procedure of the GCM SA is provided in the Methods section.

Figure 3(a) shows a top-view optical microscope image of the GCM before cutting. Only the microfibre is visible because the PMMA/graphene plane is parallel to the top-view direction. Figure 3(b,c) present lateral-view optical microscope images of the GCM before and after cutting, respectively. The photograph of the GCM together with the substrate before cutting is shown in supplementary information Fig. S1. We can see from Fig. 3(b) that the length of the GCM is approximately 210 μm. The white rectangular area in Fig. 3(b) originates from the reflection of the PMMA/graphene plane. After cutting, the width of PMMA/graphene alongside the microfibre is approximately 10 μm, as shown in Fig. 3(c). The full lateral-view optical microscope image of the GCM after cutting is included in supplementary information Fig. S2.

**Figure 4:**
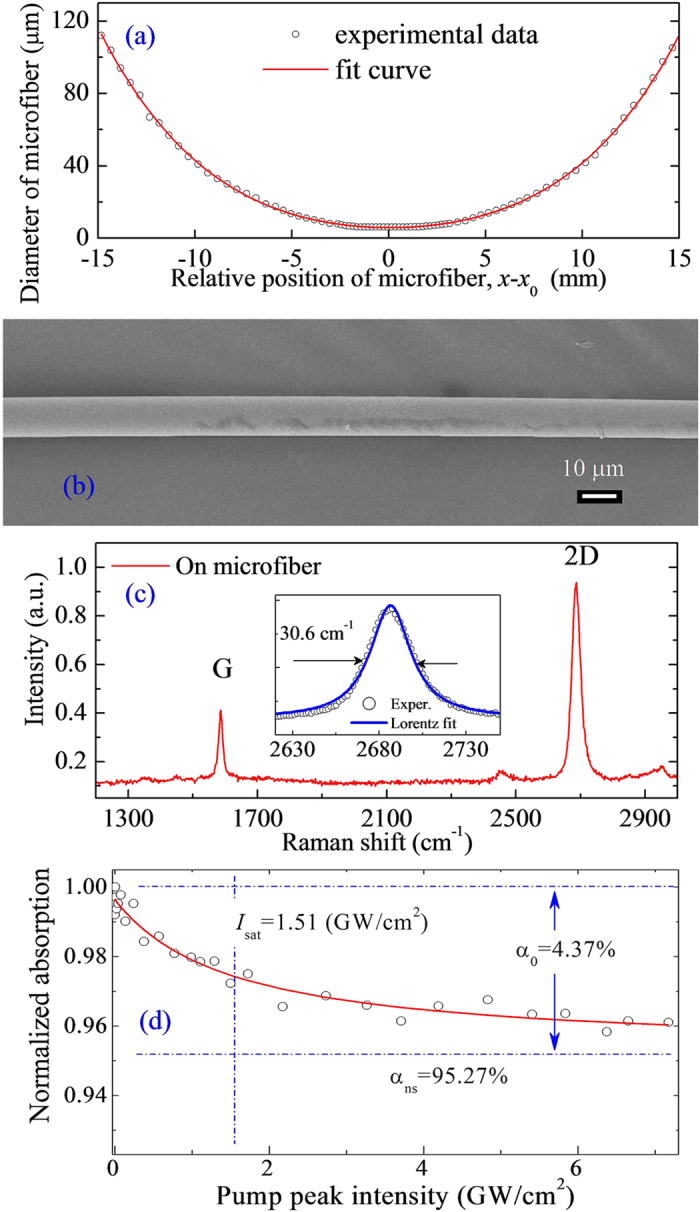
Sample characterization. (**a**) Diameter of the microfibre along the direction of propagation. *x*_0_ is the position of the microfibre at the minimum diameter. (**b**) SEM image of the GCM. Scale bar, 10 μm. (**c**) Raman spectra of the monolayer graphene film on the microfibre. (**d**) Nonlinear absorption characterization of the GCM SA. The solid curve represents a fit to the experimental data (circles).

Figure 4(a) shows the diameter of microfibre along with the propagating position, where *x*_0_ is the position of the microfibre at the minimum diameter. The circles are the experimental data, which are fitted with a polynomial function of degree 4 (red solid curve). The polynomial equation that produces the best fit is *D* = 5.8465 − 0.02548 · Δ*x* + 0.27112 · Δ*x*^2^ − 4.54967 · 10^−4^ · Δ*x*^3^ + 9.33575 · 10^−4^ · Δ*x*^4^, where *D* is the diameter of the microfibre and Δ*x* = *x* − *x*_0_ is the relative position of the microfibre. The minimum diameter of the GCM is approximately 6 μm. Figure 4(b) presents a scanning electron microscopy (SEM) image of the GCM. By comparing this image to Fig. 3, the SEM image reveals that the surface of the microfibre is smooth after the PMMA is removed. The Raman spectra of the graphene in the GCM are shown in Fig. 4(c). The symmetric Raman 2D band centred at ~2689 cm^−1^ exhibits a single Lorentzian feature with a narrow full width at half maximum (FWHM) of ~30.6 cm^−1^, and the intensity of the 2D band is much larger than that of the G band, with a 2D-to-G intensity ratio of ~3, as shown in Fig. 4(c). According to the technique of identifying single-layer graphene^31^,^32^,^33^, the microfibre is covered by monolayer graphene. Figure 4(d) shows the normalized nonlinear absorption of the GCM SA, which is experimentally measured with a homemade ultrafast fibre laser at a central wavelength of ~1550 nm and a pulse duration of ~200 fs. The experimental data are fitted using the solid curve shown in Fig. 4(d) on the basis of a simplified two-level SA model^34^,^35^,^36^. Figure 4(d) illustrates that the linear limits of the saturable absorption (α_0_), the nonsaturable absorption (α_ns_), and the saturation intensity (*I*_sat_) are approximately 4.37%, 95.27%, and 1.51 GW/cm^2^, respectively.

**Figure 5:**
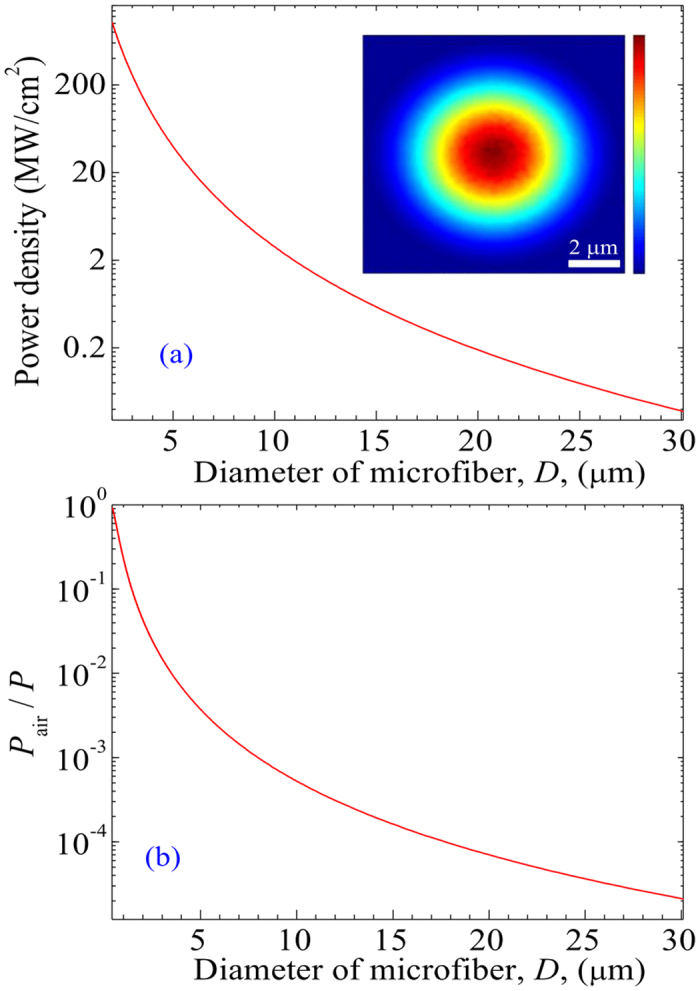
Optical properties of GCM. (**a**) Power density at the surface of the microfibre and (**b**) the energy fraction in the air (i.e., *P*_air_/*P*) for the HE_11_ mode versus the diameter of the microfibre, *D*. Inset in (**a**): the cross-sectional intensity distribution in a 10-μm diameter microfibre (calculation performed using COMSOL).

Figure 5(a,b) show the power density at the surface of the microfibre and the energy fraction in the air (i.e., *P*_air_/*P*) for the fundamental mode (i.e., HE_11_) along the diameter of the microfibre (i.e., *D*), respectively. The central wavelength of the guided wave is 1531.3 nm. The inset illustrates the cross-sectional intensity distribution in a 10-μm diameter microfibre. These figures were calculated using COMSOL. In the calculation, the peak power of the pulse wave is 200 W, which is consistent with the experimental results. One can see from Fig. 5(a) that the power density at the surface of the microfibre decreases exponentially with *D*. For instance, the power densities are approximately 20, 3, and 0.038 MW/cm^2^ for *D* = 6, 10, and 30 μm, respectively. Therefore, the light–graphene interaction is enhanced significantly in the GCM via a tightly confined evanescent field guided along the surface of the microfibre. The energy fraction in the air, *P*_air_/*P*, increases exponentially with decreasing *D*. For example, *P*_air_/*P* ≈ 22.7% and 77.6% for *D* = 1 and 0.5 μm, respectively, as shown in Fig. 5(b).

### GCM SA for ultrafast fibre lasers

**Figure 6:**
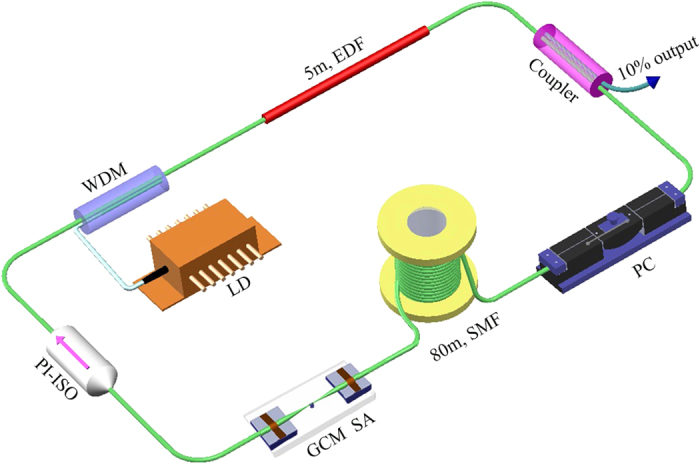
Laser setup. EDF, erbium-doped fibre; WDM, wavelength-division multiplexer; PC, polarization controller; SMF, single-mode fibre; PI-ISO, polarization-independent isolator; LD, laser diode; GCM SA, graphene-clad microfibre saturable absorber.

A schematic of the GCM-based fibre laser is shown in Fig. 6. The laser system consists of a wavelength-division multiplexer (WDM), a 5-m-long erbium-doped fibre (EDF) with 6 dB/m absorption at 980 nm, a fused coupler with an output ratio of 10%, a polarization controller (PC), an 80-m-long standard single-mode fibre (SMF), a GCM SA, a polarization-independent isolator (PI-ISO), and some fibre pigtails. The EDF provides the gain amplification for the laser system pumped by a 977-nm laser diode (LD). The PI-ISO is used to ensure the unidirectional transmission of the laser operation. PC located in front of the GCM mode-locker optimizes intracavity state of polarization (SOP), especially matching the roundtrip SOP. The total length of the laser cavity is ~110 m. The EDF and SMF have dispersion parameters of approximately −9 and 17 ps/(nm·km) at 1550 nm, respectively.

**Figure 7:**
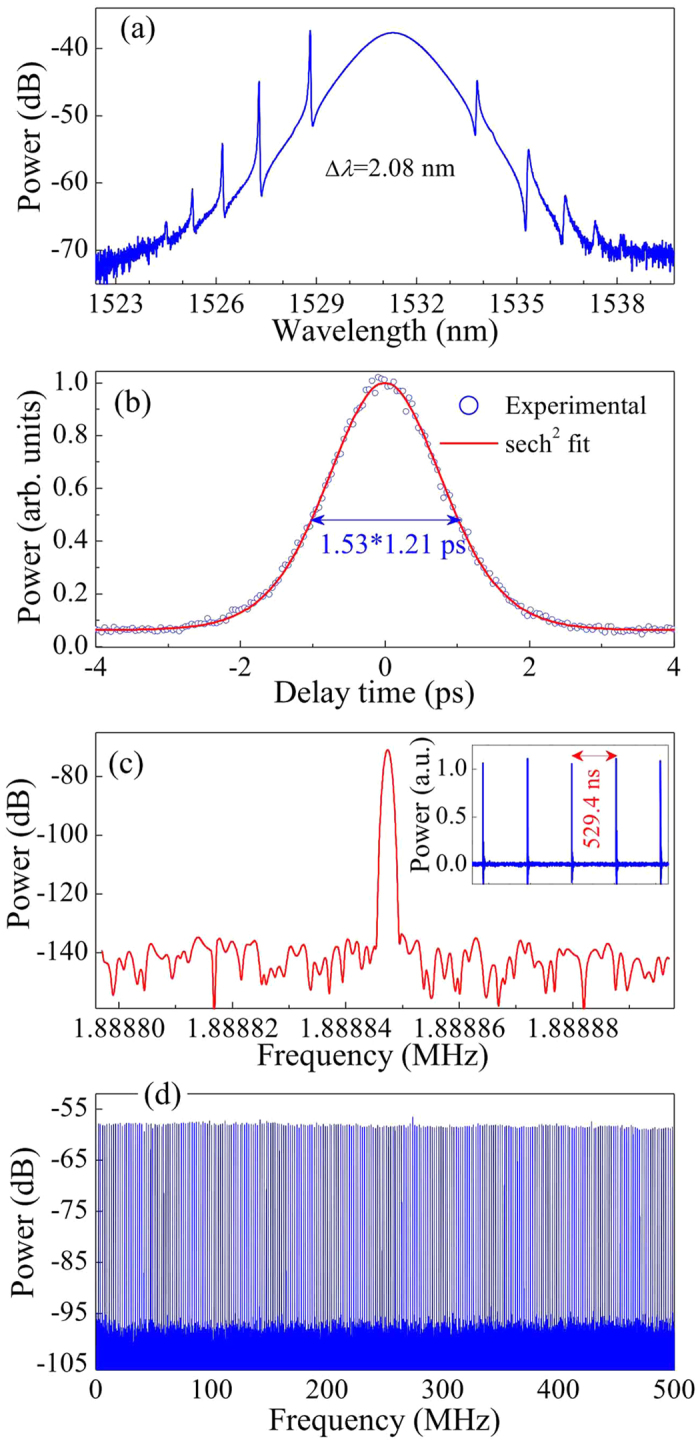
Typical laser characteristics. (**a**) Optical spectrum with a spectral resolution of 0.02 nm at pump power *P* = 27 mW. The FWHM spectral width Δ*λ* is approximately 2.08 nm. (**b**) Autocorrelation traces of the experimental data (circles) and sech^2^-shaped fit (solid curve). (**c**) Fundamental RF spectrum with a resolution of 1 Hz and a span of 100 Hz. Inset: oscilloscope trace with a separation of 529.4 ns, corresponding to 1.888847 MHz of the fundamental cavity frequency, which is independent of the pump power. (**d**) Wideband RF spectrum up to 500 MHz.

The threshold pump power for CW lasing is approximately 5 mW. Self-starting mode-locking operation starts at the proper pump power when the polarization controller is adjusted appropriately. Once the stable laser is obtained, no further polarization controller adjustment is needed. Figure 7(a) demonstrates a typical laser spectrum at a pump power of 27 mW, with a central wavelength of ~1531.3 nm. The FWHM spectral bandwidth, Δ*λ*, is 2.08 nm. The sidebands at 1527.28, 1528.81, and 1533.82 nm are typical of soliton-like pulse formations, which originate from intracavity periodic perturbations^37^. Figure 7(b) illustrates the second harmonic generation (SHG) autocorrelation traces of the experimental data and a sech^2^-shaped fit. Assuming a sech^2^ temporal profile, the deconvolution yields a pulse duration of 1.21 ps. The time bandwidth product (TBP) is 0.322, which is near the value of the transform-limited sech^2^-shaped pulses. Figure 7(c) shows that the repetition rate of the fundamental cavity frequency is 1.888847 MHz, corresponding to 529.4 ns of round-trip time (inset of Fig. 7(c)). The radio frequency (RF) spectrum in Fig. 7(c) gives a signal-to-noise ratio of >60 dB (>10^6^ contrast), indicating low-amplitude fluctuations and good mode-locking stability^38^. No spectrum modulation is observed over 500 MHz (Fig. 7(d)), thereby indicating no Q-switching instabilities.

## Discussion

In the experiments, the average power of pulses in the laser cavity is ~0.45 mW, corresponding to a peak power of ~200 W. The experimental observations demonstrate that the proposed fibre laser can generate ultrafast pulses when the diameter of GCM, *D*, is changed from ~1 to ~30 μm. We can see from Fig. 5(a) that for *D* > 30 μm, the light–graphene interaction is so weak (i.e., less than 0.038 MW/cm^2^) that the mode locking fails. When *D* is less than 1 μm, a considerable fraction of the energy of the pulse is from the microfibre, in which case slight roughness on the surface of the microfibre can cause large losses. As a result, the loss that occurs in the GCM prevents the mode-locking operation of a laser.

The experimental observations demonstrate that if the GCM-SA component is excluded from the proposed fibre laser, it fails to mode-lock; thus, the CW rather than ultrafast pulses is generated. Although the polarization effects can affect the characteristics of pulses, they hardly play such a role in emitting pulses. Both theoretical and experimental results indicate that the polarizer plays the key role in the nonlinear polarization rotation technique^39^. In this work, however, there is no polarizer that is artificially made from a polarization-sensitive isolator.

The loss that occurs in the GCM is dependent on the diameter of the taper and the length of the graphene. In this work, the loss is approximately 1 dB. Although the taper fibre is not protected, the proposed laser can work well over the long term and deliver pulses consistently in our ultraclean laboratory. Outside the ultraclean laboratory environment, the taper fibre must be protected appropriately. Because the diameter of the taper is approximately 6 μm, the damage threshold is sufficiently high.

## Methods

### Preparation of the GCM SA

The microfibre is drawn from a standard telecom optical fibre using a flame-brushing technique. The microfibre has a minimum diameter of ~6 μm, a length of ~30 mm, and a low insertion loss of 0.3 dB. The monolayer graphene is grown on Cu foil via the CVD method. After spin-coating the graphene with PMMA, the large-area PMMA/graphene/Cu film is cut into small pieces of width ~0.2 mm and length ~2 mm. The underlying Cu foil of the small piece is etched in FeCl_3_ solution for ~10 h. After the Cu is etched completely, transparent PMMA/graphene sheet floats on the surface of the solution. The PMMA/graphene sheet is then transferred into distilled water five times and is immersed for approximately 2 h each time to remove the remaining FeCl_3_ etchant. Subsequently, a microfibre, which is integrated onto a hollow glass substrate in advance, is placed under the centre of the PMMA/graphene sheet. When the microfibre is lifted together with the hollow substrate from the water, the PMMA/graphene spontaneously wraps around the microfibre (Fig. 3(b)). A femtosecond laser beam (at 790 nm with a pulse duration of 200 fs, repetition rate of 1 kHz, and power of 100 μJ) through a micro/nano platform is used to cut the PMMA/graphene to a ~10 μm width alongside the microfibre (Fig. 3(c)). After cutting, the microfibre with the PMMA/graphene is placed into acetone solution for ~20 min to remove the PMMA. Finally, the graphene wrapped around the microfibre is transferred into alcohol for ~10 min to wash away the residue of the polymer.

### Measurement method

An optical spectrum analyser (Yokogawa AQ-6370), an autocorrelator, a 6-GHz oscilloscope, a radio-frequency (RF) analyser, and a 10-GHz photodetector are used to measure the laser output performance.

## Additional Information

**How to cite this article**: Liu, X. M. *et al*. Graphene-clad microfibre saturable absorber for ultrafast fibre lasers. *Sci. Rep.* **6**, 26024; doi: 10.1038/srep26024 (2016).

## Supplementary Material

Supplementary Information

Supplementary Information

## Figures and Tables

**Figure 1:**
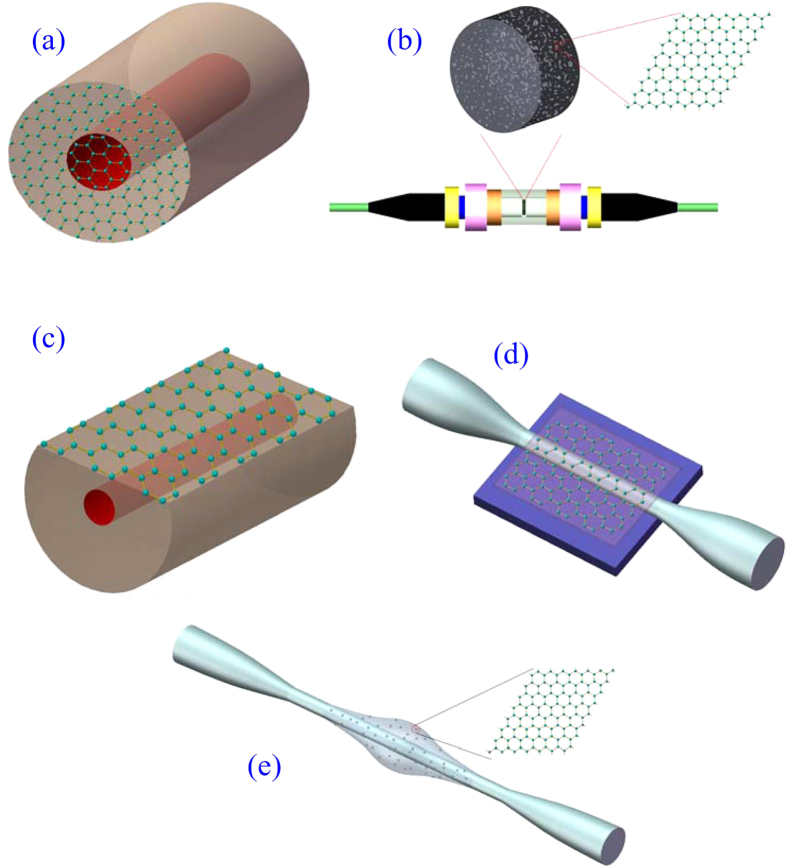
Schematic illustration of graphene-based SAs with (**a**) a graphene film coating on a pinhole (red), (**b**) a graphene–PVA nanocomposite film integrated between a pair of fibre connector ends, (**c**) a graphene film coating on the D-shaped fibre and (**d**) on the microfibre, and (**e**) a graphene/polymer composite embedded on the microfibre.
