# Supplementary material for: Graphene-clad microfibre saturable absorber for ultrafast fibre lasers
Source: Sci Rep. 2016 May 16;6:26024. doi: 10.1038/srep26024 (PMC4867430; doi:10.1038/srep26024)
Supplement: Supplementary Information [file srep26024-s1.doc]

**Supporting Information**

Graphene-clad microfiber saturable absorber for ultrafast fiber lasers

X. M. Liu, ^1,*^ H. R. Yang, ^1^ Y. D. Cui, ^1^ G. W. Chen, ^1^ Y. Yang, ^1^ X. Q. Wu, ^2^ X. K. Yao, ^1^ D. D. Han, ^1^ X. X. Han, ^1^ C. Zeng, ^1^ J. Guo, ^1^ W. L. Li, ^1^ G. H. Cheng, ^1^ and L. M. Tong ^2^

^1^ State Key Laboratory of Transient Optics and Photonics, Xi’an Institute of Optics and Precision Mechanics, Chinese Academy of Sciences, Xi’an 710119, China

*^2^ State Key Laboratory of Modern Optical Instrumentation, Department of Optical Engineering, Zhejiang University, Hangzhou 310027, China*

*Corresponding author: [liuxueming72@yahoo.com](mailto:liuxueming72@yahoo.com)

**Cutting procedure of the graphene-clad microfiber**

A femtosecond laser micromachining system is employed to cut the graphene-clad microfiber (GCM). The femtosecond laser operates at the central wavelength of ~790 nm with the pulse duration of ~200 fs, the repetition rate of ~1 kHz, and the pulse energy of ~100 μJ. The GCM is placed on a hollow glass substrate, as shown in Figure S1, and then fixed on the micro-positioning stage. The GCM has an about 1-mm-long rectangular PMMA/graphene tail suspended off the microfiber before the cutting procedure (as depicted in Fig. 3(b)). The laser beam is focused onto the PMMA/graphene. The cutting procedure is performed by sweeping the PMMA/graphene from left to right along the microfiber with proper speed. About ~10 s later, the rectangular PMMA/graphene tail is cut off from the microfiber, left about 10 μm width alongside the fiber, which is displayed in Figure S2.


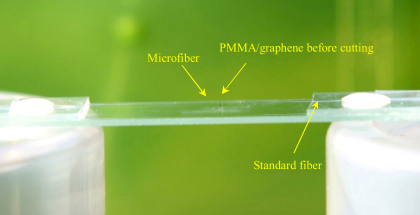


Figure S1. Photograph of the GCM fixed on the hollow glass substrate. A rectangular PMMA/graphene film is suspended on the center of microfiber.

Figure S2. Optical microscope image of the GCM after the cutting procedure.

The supplementary video (CutGraphene.swf) shows the detailed cutting procedure of Fig.3.
